# NK cells control HIV‐1 infection of macrophages through soluble factors and cellular contacts in the human decidua

**DOI:** 10.1186/s12977-016-0271-z

**Published:** 2016-06-06

**Authors:** H. Quillay, H. El Costa, M. Duriez, R. Marlin, C. Cannou, Y. Madec, C. de Truchis, M. Rahmati, F. Barré-Sinoussi, M. T. Nugeyre, E. Menu

**Affiliations:** Unité de Régulation des Infections Rétrovirales, Institut Pasteur, Paris, France; Université Paris Diderot, Sorbonne Paris Cité, Cellule Pasteur, Paris, France; Sorbonne Universités, UPMC Univ Paris 06, INSERM U1135, Centre d’Immunologie et des Maladies Infectieuses (CIMI), Persistent Viral Infections (PVI) Team, Paris, France; Immunology of Viral Infections and Autoimmune Diseases/IDMIT Infrastructure, CEA/DRF/iMETI/Division of Immuno-Virology, Université Paris Sud, Inserm U1184, Fontenay-aux-Roses, France; Vaccine Research Institute (VRI), Créteil, France; Unité d’Epidémiologie des Maladies Emergentes, Institut Pasteur, Paris, France; Gynecology-Obstetrics Service, A. Béclère Hospital, AP-HP, Clamart, France; Gynecology-Obstetrics Service, Pitié Salpêtrière Hospital, AP-HP, Paris, France

**Keywords:** NK cells, Macrophages, Decidua, HIV-1, IFN-γ, Control, Mother-to-child transmission

## Abstract

**Background:**

During the first trimester of pregnancy, HIV-1 in utero transmission is rare despite the permissivity of the placenta and the decidua (the uterine mucosa during pregnancy) to infection. In the decidua from the first trimester of pregnancy, macrophages (dMs) are the HIV-1 main target cells. Decidual natural killer (dNK) cells account for 70 % of decidual leukocytes. They display distinct phenotype and functions compared to peripheral NK cells. At the periphery, NK cells are involved in the control of HIV-1 infection. In this study, we investigate whether human decidual natural killer (dNK) cells control dM HIV-1 infection.

**Results:**

Autologous cocultures of infected dMs with dNK cells reveal that dNK cells strongly inhibit dM HIV-1 infection. The addition of dNK cells to dMs at different times after infection suggests that the control occurs before the complete establishment of the infection. Double chamber cocultures show that cellular contacts are necessary for an optimal control of infection. Nevertheless, soluble factors secreted by dMs and dNK cells in double chamber cocultures partially inhibit dM HIV-1 infection, indicating that soluble factors have also a role in the control of infection. IFN-γ secretion is increased in infected and uninfected cocultures. We show that IFN-γ is involved in the control of dM HIV-1 infection by dNK cells.

**Conclusions:**

These results demonstrate that human dNK cells inhibit efficiently HIV-1 infection in dMs in vitro, and highlight the role of innate immune determinants in the control of HIV-1 transmission.

**Electronic supplementary material:**

The online version of this article (doi:10.1186/s12977-016-0271-z) contains supplementary material, which is available to authorized users.

## Background

Mucosae are the preferential portal for Human Immunodeficiency Virus type 1 (HIV-1) entry in the body. It is thus important to identify the immune responses necessary for an efficient control of HIV-1 transmission in the mucosa. The study of models of natural protection against HIV-1 transmission at a mucosal level should help to identify mechanisms of control. The materno-fetal interface during the first trimester of pregnancy is one of these models.

During the first trimester of pregnancy, HIV-1 in utero transmission is relatively rare [1], and may occur through the infection of cells at the materno-fetal interface. The main materno-fetal interface is made up of the *decidua basalis* (the uterine mucosa during pregnancy) and the placenta. In vitro, the decidua and the placenta are permissive to HIV-1 infection [2]. In the decidua, macrophages (CD14^+^ cells, dMs) are the main HIV-1 R5 target cells [3]. The low frequency of HIV-1 in utero transmission, despite the permissivity of the placenta and the decidua to infection, indicates that there is a control of HIV-1 infection at the materno-fetal interface. Several studies have been conducted on the placenta. It has been shown that some cytokines and chemokines, antibodies and co-infections influence HIV-1 infection of placental cells [2]. Fewer studies have been performed on the control of HIV-1 infection in the decidua. Decidual cell culture supernatants decrease HIV-1 infection of decidual mononuclear cells by inhibiting HIV-1 entry [4]. Decidual cell culture supernatants contain the chemokines CCL3 and CCL4, which inhibit HIV-1 infection by binding the CCR5 HIV-1 co-receptor. However, the control by decidual cell culture supernatant is partial, suggesting that other mechanisms exist [4].

The decidua is made up of 40 % of leucocytes, among which 20 % are macrophages and 70 % are Natural Killer (dNK) cells during the first trimester of pregnancy [5]. NK cells are a major component of the innate immune system. At the periphery several studies have highlighted the role of NK cells in the control of HIV-1 infection [6]. NK cells from HIV-1 long-term non-progressors (individuals infected with HIV-1, with a detectable viral load, but a CD4 T cell count >600 cells/μL in absence of antiretroviral therapy) have a higher lytic and secretory activity than NK cells from uninfected individuals [7, 8]. Moreover, in individuals exposed to HIV-1 through the use of injectable drugs but who remains uninfected, a high lytic and secretory NK cell activity has been observed either after in vitro activation or without stimulation [9]. In these studies, one of the main induced soluble factors was the IFN-γ, an antiviral soluble factor. In vitro, it has been shown that the CD85j^+^ NK cells subpopulation inhibits HIV-1 infection of monocytes-derived dendritic cells (MDDC) [10].

dNK cells have a different phenotype from peripheral NK cells [11, 12]. dNK cells are CD56^superbright^ CD16^neg^, and they constitutively express the activation marker CD69. dNK cells produce large amounts of cytokines, chemokines and angiogenic factors, and they are weakly cytotoxic in a normal pregnancy. However, dNK cell cytotoxic activity can be induced by activating signal such as cytokines or activation of specific receptors like NKp46 [13, 14]. dNK cells are important to achieve a successful pregnancy. In fact, they regulate angiogenesis and trophoblast invasion [15, 16]. A recent study show that, in vitro, dNK cells are able to kill fibroblasts infected by the human cytomegalovirus (HCMV) [17]. This study suggests that dNK cells could protect the fetus against the transmission of pathogens from the mother. Nevertheless, the role of dNK cells in the control of HIV-1 infection at the materno-fetal interface is unknown.

During the first trimester of pregnancy, dMs and dNK cells are in close contact in the decidua [13]. We have previously shown that dMs and dNK cells could interact through cellular contacts. In fact dMs, from the first trimester of pregnancy, express the NKG2D ligand ULBP1 and the CD85j and KIR2DL4 ligand HLA-G [18]. Moreover, dNK cells and dMs could interact through soluble factors. In fact dMs and dNK cells secrete soluble factors involved in the immune crosstalk [19]. Some of the soluble factors secreted by dMs and dNK cells have a role in the control of HIV-1 infection. For example, dNK cells secrete IFN-γ, and this secretion is significantly increased by TLR stimulation [19].

The aim of this study was to investigate the role of dNK cells in the control of dM infection by HIV-1. We have shown that dNK cells control HIV-1 infection of dMs in vitro through cellular contacts and soluble factors and that IFN-γ is involved in this control.

## Results

### Decidual NK cells control HIV-1 infection of decidual macrophages

**Fig. 1:**
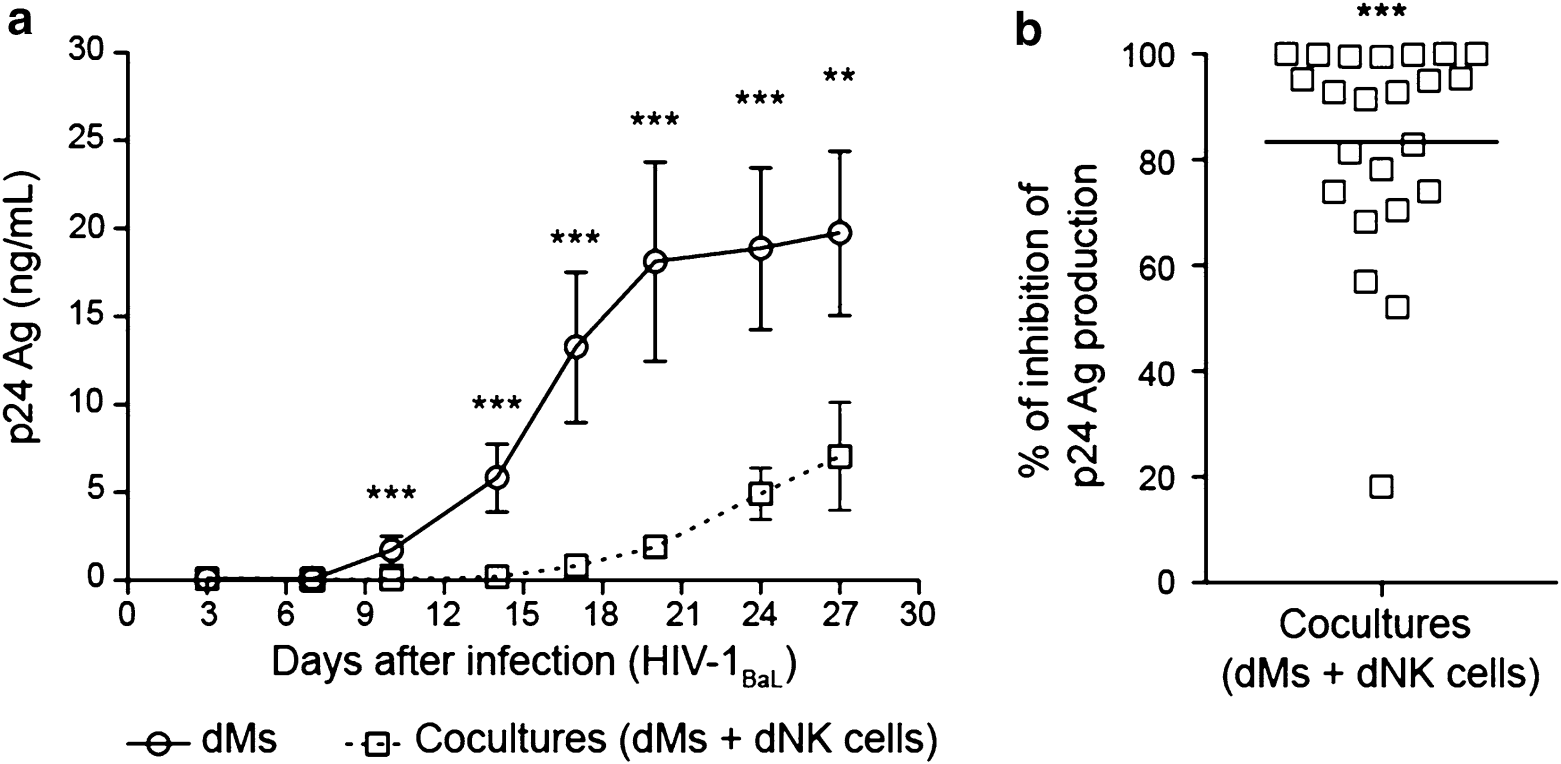
Control of dM HIV-1 infection by dNK cells. dMs were infected with HIV-1_BaL_ at an MOI of 10^−3^. dNK cells were added to dMs after the infection at a ratio 1 dM:5 dNK. Viral production was followed by the quantification of the p24 Ag in the supernatants collected every 3 or 4 days. **a** The mean of the p24 Ag concentration in dM supernatants (*circle symbol* and *full line*) and in coculture supernatants (*square symbol* and *dotted line*) of 23 donors is displayed over time. *Bars* indicate standard error of the mean. **b** The percentage of inhibition of the p24 Ag production was calculated at day 20 post-infection. The mean of 23 donors is displayed on the graph. *Each symbol* represents one individual donor. The viral production in dMs cultured alone and in coculture with dNK cells were compared. The sign-rank test for paired data was used. **p < 0.005; ***p < 0.0005

Freshly purified primary dMs were infected with HIV-1_BaL_ (R5) at an MOI of 10^−3^, and autologous dNK cells were added to dMs at a ratio 1 dM:5 dNK. Viral production was monitored over time by the quantification of the HIV-1 p24 antigen (Ag) in culture supernatants. The viral production was lower and delayed in coculture compared to dMs in culture alone (Fig. 1a). At day 20 post-infection (end of the exponential phase of dM viral production), the viral production was totally inhibited for some donors, whereas for other donors the inhibition was partial (mean of 83.4 %, minimum 18.2 % and maximum 100 %, *p* < 0.0001, 23 donors) (Fig. 1b).

In the decidua, the dM:dNK ratio varies from 1:3 to 1:5. Cocultures have also been performed at a ratio 1:3. As we have observed with the 1:5 ratio (Fig. 1), the viral production was lower and delayed in coculture compared to dMs in culture alone (data not shown). In the decidual samples used in these studies, the mean of the dM:dNK ratio was 1:5 (mean of 53 donors).

These results show that, in vitro, dNK cells control very efficiently HIV-1 infection of dMs.

### dNK cells do not control efficiently dM HIV-1 infection when they are added to dMs late after infection

To determine when the control of dM HIV-1 infection occurs, dNK cells were added to dMs at different times, before infection, 1, 3 or 16 h after infection. Samples from different donors were used and for each sample all conditions were performed. The donors used in this experiment were not the same as the one used for the experiment displayed on Fig. 1.

**Fig. 2:**
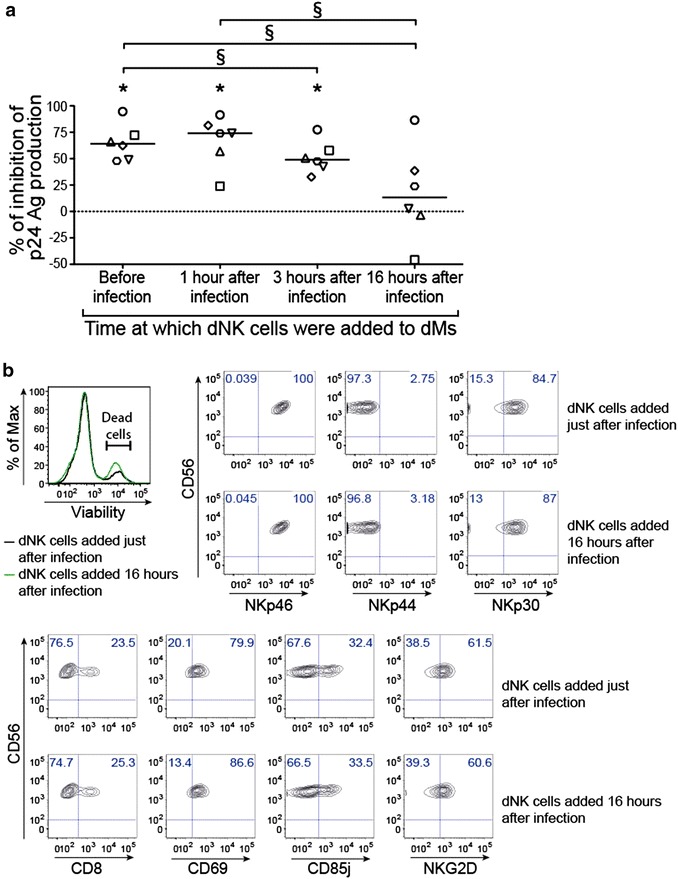
Inhibition of dM infection by dNK cells added at different times. **a** dMs were infected with HIV-1_BaL_ at an MOI of 10^−3^. dNK cells were added to dMs before infection, 1, 3 or 16 h after infection, at a ratio 1 dM:5 dNK. For each donor, all conditions were performed. Viral production was followed by the quantification of the p24 Ag in the supernatants. The percentage of inhibition of the p24 Ag production was calculated at day 20 post-infection. *Each symbol* represents one individual donor. The medians of 6 donors are displayed on the graph. The sign-rank test for paired data was used. ^§^ *p* = 0.031 between the different coculture conditions (dNK cells added to dMs before, 1, 3 or 16 h after infection). *p = 0.031 between dMs alone and in coculture with dNK cells. **b** The viability of dNK cells added to dMs just after (*black*) or 16 h after dM infection (*green*) was analysed by flow cytometry, with a viability dye labelling the dead cells. A representative FACS histogram gated on CD56^+^ cells is shown. The expression of CD8, CD69, CD85j, NKG2D, NKp46, NKp44 and NKp30 were analysed on dNK cells just after or 16 h after dM infection by flow cytometry staining. Flow cytometry graphs gated on CD56^+^ cells from a representative sample are displayed

The percentage of inhibition of dM viral production in the different coculture conditions was calculated for 6 donors at the end of the exponential phase of dM viral production, at day 20 post-infection (Fig. 2a). When dNK cells were added before, 1 or 3 h after infection, a significant inhibition of infection was observed for the 6 samples (median of 64.2, 74 and 49 % respectively, 6 donors) (Fig. 2a). When dNK cells were added 3 h after infection, the percentage of inhibition was significantly lower than when dNK cells were added before infection (*p* = 0.031). When dNK cells were added 16 h after infection, the percentage of inhibition was significantly lower (median of 13.3 %) than when dNK cells were added before or 1 h after infection (*p* = 0.031). In this condition, we observed a low inhibition (2 donors), no inhibition (2 donors) or even an increase of the viral production (1 donor). The FDR correction estimates the proportion of discoveries that are false among all discoveries when multiple comparisons are conducted [20]. By using the False Discovery Rate Correction, out of the 6 significant differences, 0.3 (q-value = 0.05) could be expected to be significant by chance. The differences between the percentage of inhibition of the four experimental conditions at days 24 and 27 post-infection were similar to the one observed at day 20 (data not shown).

The viability and the phenotype (Fig. 2b) of dNK cells added just after or 16 h after infection were analysed by flow cytometry. The viability of dNK cells was similar in both conditions (Fig. 2b). The percentages of expression of CD8, CD69, CD85j, NKG2D, NKp46, NKp44 and NKp30 were similar among dNK cells added just after or 16 h after infection (Fig. 2b). These results indicate that the decrease of the percentage of inhibition when dNK cells were added 16 h after infection is not due to a higher mortality or to a decrease of dNK cell activation after the 16 h of storage.

In conclusion, the control of HIV-1 infection is more efficient when dNK cells are added before infection or at an early time after infection.

### The control of dM HIV-1 infection by dNK cells is mediated by cellular contacts and soluble factors

To define if cellular contacts and/or soluble factors are necessary for the control of dM HIV-1 infection, double chamber cocultures and cocultures were performed in parallel for each sample. The donors used in this experiment were not the same than the one used for the experiment displayed on Figs. 1 and 2.

**Fig. 3:**
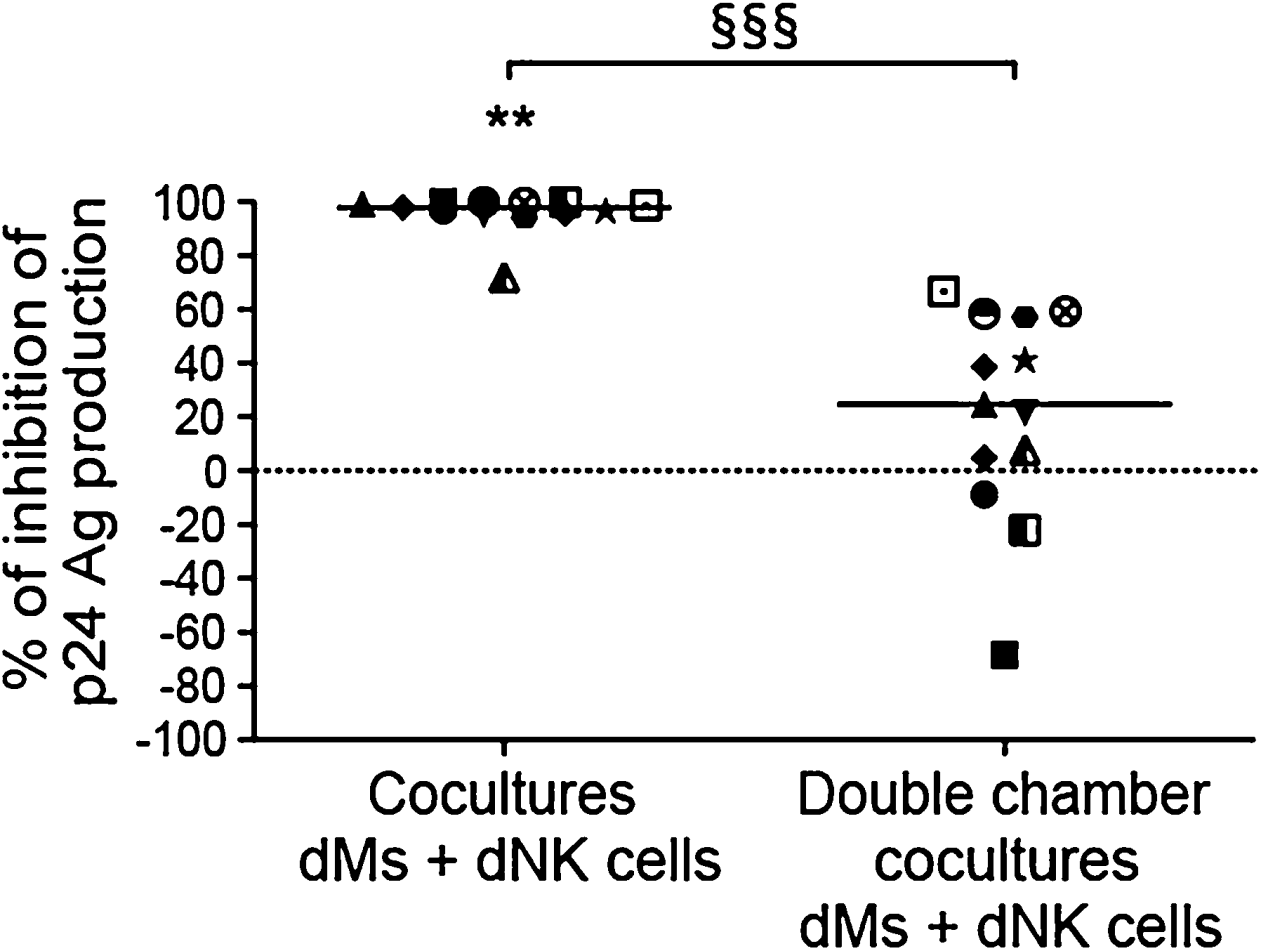
Inhibition of dM infection in cocultures or in double chamber cocultures. dMs were infected with HIV-1_BaL_ at an MOI of 10^−3^. Cocultures were performed in the same well (coculture) or separately in a double chamber system (double chamber coculture), after dM infection, at a ratio 1 dM:5 dNK. For each donor, both conditions were performed. Viral production was followed by the quantification of the p24 Ag in the supernatants. The percentage of inhibition of the p24 Ag production was calculated at day 20 post-infection. *Each symbol* represents one donor. The medians of 13 donors are depicted on the graph. The sign-rank test for paired data was used. ^§§§^ *p* = 0.0002 between cocultures and double chamber cocultures.**p = 0.002 between dMs alone and in cocultures with dNK cells

The percentage of inhibition of dM infection in coculture and double chamber coculture was calculated for 13 donors at the end of the exponential phase of dM viral production, at day 20 post-infection (Fig. 3). When dNK cells and dMs were cocultured in the same well, the percentage of inhibition was higher than when dNK cells and dMs were separated by a double chamber system (median of 97.8 and 24.8 % respectively, *p* = 0.0002, 13 donors). When dMs and dNK cells were cocultured in the same well, the viral production was almost undetectable. For 3 donors among 13, when cells were cultured separately, an increase of the viral production was observed compared to dM viral production. The difference between the percentage of inhibition of the two experimental conditions at day 24 and 27 post-infection were similar to the one observed at day 20 (data not shown).

These results demonstrate that cellular contacts between dMs and dNK cells are necessary for an optimal control of HIV-1 infection. For 8 donors among 13, a partial inhibition of HIV-1 infection is observed when dMs and dNK cells are cultured separately indicating also that soluble factors are involved.

### IFN-γ is secreted in coculture of dMs and dNK cells

**Fig. 4:**
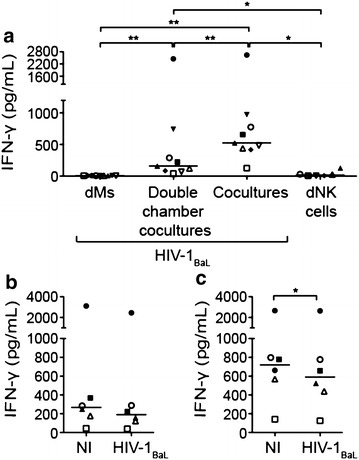
IFN-γ concentration in the culture supernatants. **a** The IFN-γ concentrations in 48 h supernatants of infected dMs, infected double chamber cocultures, infected cocultures and dNK cells are depicted on the graph in pg/mL (9 donors, except for dNK cell supernatants, 6 donors). The IFN-γ concentrations in supernatants of **b** uninfected (NI) and infected (HIV-1_BaL_) double chamber cocultures, **c** uninfected (NI) and infected (HIV-1_BaL_) cocultures are displayed on the graph in pg/mL (6 donors). The medians are displayed. The sign-rank test for paired data was used. *p = 0.031; **p = 0.004

Given the IFN-γ antiviral activity, its concentration was analysed in the supernatants of infected dMs, infected cocultures, infected double chamber cocultures and dNK cells. Supernatants were harvested 48 h post-infection. IFN-γ was undetectable in infected dM supernatants (Fig. 4a, 9 donors). The concentration of IFN-γ was increased in infected coculture and double chamber coculture supernatants, but less in double chamber coculture supernatants than in coculture supernatants (median of 159.7 pg/mL and 525.4 pg/mL, respectively, *p* = 0.004, 9 donors). In dNK cell supernatants, the IFN-γ concentration was significantly lower than the one in coculture and double chamber coculture supernatants (median of 15.6 pg/mL, both *p* = 0.031, 6 donors). These observations indicate that the IFN-γ secretion is increased by cellular contact between dMs and dNK cells, and by soluble factors secreted by dMs and dNK cells in coculture.

The IFN-γ concentration was also analysed in the supernatants of uninfected cocultures and double chamber cocultures. For each donor, all conditions were performed. IFN-γ concentration was in the same order of magnitude in 48 h supernatants of uninfected compared to infected double chamber cocultures (Fig. 4b), and uninfected compared to infected cocultures (Fig. 4c). IFN-γ concentration was slightly higher in uninfected double chamber coculture supernatants than in infected double chamber coculture supernatants, and in uninfected coculture supernatants than in infected coculture supernatants (median of 267.2 and 191.1 pg/mL for double chamber cocultures, respectively; 720.3 and 591.3 pg/mL for cocultures, respectively, *p* = 0.031, 6 donors). As in infected dM supernatants, IFN-γ was undetectable in uninfected dM supernatants as previously reported [19].

In conclusion, IFN-γ concentration is increased in infected and uninfected coculture and double chamber coculture supernatants.

### IFN-γ participates to the control of dM HIV-1 infection by dNK cells

**Fig. 5:**
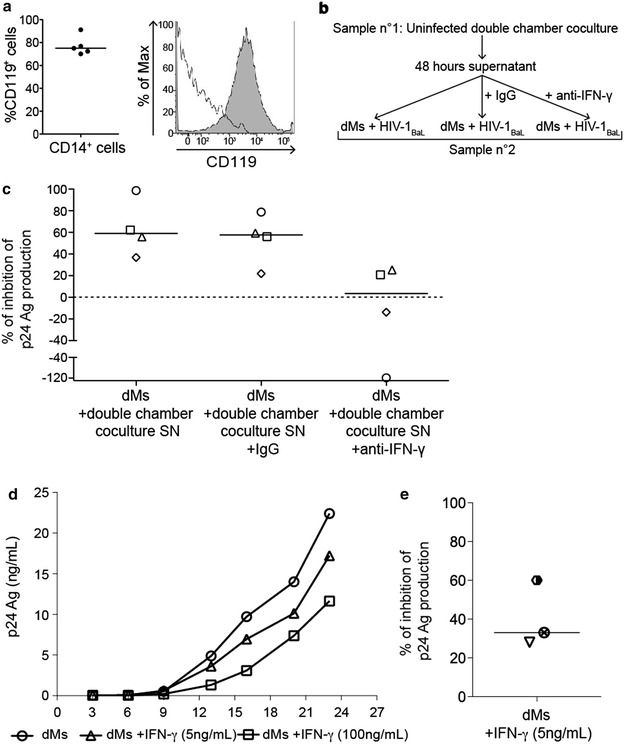
Role of IFN-γ in the control of dM infection by dNK cells. **a** CD119 expression was analysed on dMs by flow cytometry. Values depicted on the graph are the percentages of expression of CD119 among dMs from 5 different donors. The median is also displayed. A representative FACS histogram gated on CD45^+^ CD14^+^ cells is shown (filled grey profile, anti-CD119 antibody and white profile, control). **b** Representation of the experimental system. Double chamber coculture supernatant (SN) from one donor (donor no. 1) were treated or not with an IgG isotype control or an anti-IFN-γ antibody. dMs from another donor (donor no. 2) infected with HIV-1_BaL_ at an MOI of 10^−3^ one hour before, were then incubated during 3 days with these supernatants. Viral production was followed by the quantification of the p24 Ag in the supernatants. Each supernatant was incubated with dMs from a different donor. **c** The percentage of inhibition of the infection was calculated at the time point corresponding to the largest dM viral production inhibition. For each donor, all conditions were performed. Each symbol represents one donor. The medians of the percentage of inhibition from 4 donors are displayed. **d** and **e** dMs were infected with HIV-1_BaL_ at an MOI of 10^−3^, treated with recombinant human IFN-γ (5 or 100 ng/mL) during 3 days and then left in culture in an IFN-γ free medium. Viral production was followed as previously described. **d** The viral production kinetics for a representative donor is displayed over time. **e** The percentage of inhibition of the infection of dMs treated with 5 ng/ml of IFN-γ was calculated at the time point corresponding to the largest dM viral production inhibition. The median of the percentage of inhibition from 3 donors is displayed

To determine whether IFN-γ could be involved in the control of dM HIV-1 infection, the expression of CD119, i.e. the IFN-γ receptor α-chain, was first analysed on dMs. Flow cytometry analyses showed that CD119 was expressed on dMs (median of 75 %, 5 donors) suggesting that IFN-γ could have an effect on dMs (Fig. 5a).

IFN-γ blocking experiments were then performed. For each experiment, 48-h supernatants of uninfected double chamber coculture (dM and dNK cells) were left untreated, or were treated with an anti-IFN-γ antibody or an IgG isotype control (Fig. 5b). Then dMs from another donor were incubated with these supernatants, just after HIV-1 infection. For each donor, all conditions were performed. The supernatants from 4 different donors were incubated with dMs from 4 different donors. The donors used for this experiment were not the same than the one used for the experiment displayed on Figs. 1, 2 and 3. The percentage of inhibition of the infection was calculated at the time point corresponding to the largest dM viral production inhibition. For all donors, we have observed an inhibition of dM viral production by double chamber coculture supernatants and by double chamber coculture supernatants treated with the isotype control (Fig. 5c). The percentages of inhibition of dMs incubated with supernatants treated or not with an IgG isotype control were similar (median of 57.7 and 59.0 %, respectively, 4 donors). When dMs were incubated with supernatants treated with an anti-IFN-γ antibody, the percentage of inhibition decreased in all samples available (median of 3.5 %) and the *p* value, obtained by a permutation test, was the smallest that could be achieved given the sample size available (*p* = 0.0625). For two donors out of four, the viral production of dMs incubated with the anti-IFN-γ antibody treated supernatants was even higher than viral production of dMs alone.

To confirm the role of IFN-γ in the control of dM HIV-1 infection, infected dMs were treated just after infection with recombinant human IFN-γ at different concentrations. The donors used for this experiment were not the same as the one used for the experiment displayed on Figs. 1, 2, 3 and 5c. The viral production kinetics for a representative donor is shown in Fig. 5d. The viral production of untreated dMs was higher than the one of dMs treated with IFN-γ (1.4 and 1.9 time higher for 5 and 100 ng/mL, respectively, at day 20 post-infection). The viral production of dMs treated with IFN-γ at 100 ng/mL was lower than the one of dMs treated with IFN-γ at 5 ng/mL. When dMs were treated with IFN-γ at 0.5 ng/mL, a lower inhibition was observed (data not shown). dM viral production inhibition by IFN-γ was dose dependent. The percentage of inhibition of the infection of dMs treated with IFN-γ at 5 ng/mL was calculated at the time point corresponding to the largest dM viral production inhibition. For all donors, IFN-γ (5 ng/mL) inhibited dM viral production (median of the percentage of inhibition 33 %, 3 donors) (Fig. 5e).

In conclusion, soluble factors secreted by dMs and dNK cells cocultured separately, are able to inhibit partially dM HIV-1 infection. The inhibition is lower or is overcome when IFN-γ is blocked in these supernatants. Moreover, IFN-γ treatment of dMs decreases the viral production of dMs. These results indicate that IFN-γ plays a role in the control of HIV-1 infection of dMs by dNK cells.

## Discussion

In the present study, we report for the first time that, in vitro, dNK cells control HIV-1 infection of dMs, the main HIV-1 target cells in the decidua. At the periphery, it has been shown that NK cells control HIV-1 infection of MDDC in vitro [10], and that NK cells from HIV-1 long-term non-progressors and HIV-1 exposed uninfected individuals could play a role in the control of HIV-1 infection [7–9, 21]. Nevertheless, dNK cells and peripheral NK cells have a different phenotype and dNK cells have specific functions [11, 12, 15]. Our study shows that NK cells from a mucosa, the decidua, can control HIV-1 infection.

Since, mother to child transmission of HIV-1 is rare during the first trimester of pregnancy [1], dNK cells could participate to the control of in utero transmission of HIV-1. It has been previously suggested that dNK cells could control HCMV in utero transmission [17]. These data highlight the fact that, besides their important role of builder in pregnancy, dNK cells could limit pathogen transmission to the fetus.

We have shown that when dNK cells are added to dMs several hours (3–16 h) after infection, the control of infection is reduced. This result was not due to a decrease of dNK cell viability or to a modification of dNK cell phenotype during the few hours before dNK cells were added to dMs. For two samples, dNK cells were added to dMs 24 h after infection and no inhibition of dM viral production was observed (Additional file 1: Fig S1). These results confirm that dNK cells control efficiently dM HIV-1 infection. The cell concentration was higher in coculture than when the dMs were culture alone, however since the inhibition of the infection in coculture was not seen anymore at the latest time point, it indicates that the control is not due to the culture experimental conditions. Moreover, these results demonstrate that the control of dM infection by dNK cells occurs before the complete establishment of the infection. In vivo, during a healthy pregnancy, dM and dNK cells are in close contact in the decidua [13], suggesting that HIV-1 infection of dMs could be tightly controlled.

The control of infection was more efficient when dNK cells and dMs were in coculture in the same well, indicating that cellular contacts between dMs and dNK cells are necessary for an optimal control of dM HIV-1 infection. As HIV-1 has developed evasion strategies against NKG2D mediated NK cell activation, NKG2D has been suggested to play a role in the control of HIV-1 infection [22]. We have previously shown that the NKG2D ligand ULBP1 is expressed on freshly purified dMs [18]. To determine if NKG2D has a role in the control of dM HIV-1 infection by dNK cells, NKG2D and NKG2D ligands have been blocked in the coculture experiments. Even if the blocking of NKG2D or NKG2D ligands overcome the inhibition of dM HIV-1 infection by dNK cells for some donors, this result was not significant. At the periphery, the blocking of CD85j ligands raises the control of HIV-1 replication in MDDC by CD85j^+^ NK cells [10]. In the decidua, the blocking of CD85j ligands on dMs has no effect on the control of infection by dNK cells in the tested donors (Additional file 2: Fig S2 for NKG2D and CD85j blocking experiments). Other receptors could be involved in the control of dM infection by dNK cells. In particular, the activation of dNK cells through the engagement of NKp30 induce the secretion of IFN-γ by dNK cells [14]. As we have shown that IFN-γ participates to the control of dM HIV-1 infection by dNK cells, NKp30 could have a role in the control. Further experiments are necessary to identify the receptors and the ligands involved in the control of dM HIV-1 infection by dNK cells.

The partial control of dM HIV-1 infection by dNK cells when both cells are cultured separately in a double chamber system, indicates that soluble factors secreted by dNK cells and dMs, inhibit dM HIV-1 infection. This is confirmed by the partial inhibition of dM infection by double chamber coculture supernatants.

IFN-γ was detected in coculture, double chamber coculture and dNK cell supernatants. The concentrations of IFN-γ were higher in coculture indicating that the interactions between dNK cells and dMs, through soluble factors and through cellular contacts, increase the IFN-γ secretion. IFN-γ secretion increases quickly when dNK cells were in coculture with dMs (peak of secretion between 12 and 48 h, data not shown). Among the 25 soluble factors analysed by the Luminex assay, IFN-γ was one of the soluble factors whose secretion was most strongly increased in coculture. IFN-γ was undetectable in dM supernatants but was detected in dNK cell supernatants, suggesting that IFN-γ is specifically secreted by dNK cells. Moreover, we have previously reported that the stimulation of TLR2, TLR3, TLR4, TLR7/8 and TLR9 on dNK cells and dMs induce the secretion of IFN-γ by dNK cells but not by dMs [19].

It has been previously reported that, in long-term non-progressors, the percentage of peripheral IFN-γ^+^ NK cells is inversely correlated to the viral load [7]. It has also been shown that HIV-1 replication is decreased in monocyte-derived macrophages (MDM) differentiated with IFN-γ [23]. IFN-γ treatment of MDMs increases the expression of some restriction factors and anti-HIV miRNA ^22^. In the decidua, we have recently reported that IFN-γ addition to cultured dMs maintains the low permissivity of dMs, while dMs become more permissive to HIV-1 infection in cytokine-free culture [24]. IFN-γ inhibits HIV-1 infection by mechanisms involving the cyclin-dependent kinase inhibitor p21 Cip1/Waf1. By blocking the IFN-γ in double chamber coculture supernatants and by incubating infected dMs with IFN-γ, we confirm here that IFN-γ has a role in the inhibition of dM HIV-1 infection.

dNK cells secrete high level of IFN-γ during pregnancy. IFN-γ has a role in the regulation of trophoblast invasion and in vascular remodeling [25–27]. HIV-1 infection of dMs has a low effect on IFN-γ secretion by dNK cells in coculture with dMs. This result suggests that the control of dM infection by dNK cells through IFN-γ secretion might not be specific of HIV-1 infection. Altogether, these data are in favor of a role of IFN-γ secreted by dNK cells in the control of HIV-1 transmission at the materno-fetal interface. IFN-γ could also limit the in utero transmission of other pathogens than HIV-1, as it has an anti-viral activity against other viruses. Beside its important role to achieve a successful pregnancy, IFN-γ could be a key player to ensure protection of the fetus against infection.

For half of the samples, the blocking of IFN-γ overcomes only partially the inhibition of infection by dNK cells, suggesting that other soluble factors may be involved in the control of HIV-1 infection. The chemokines CCL4 and CCL5 have been detected in double chamber coculture supernatants (Additional file 3: Fig S3). These chemokines are known to inhibit HIV-1 entry by binding the CCR5 coreceptor [28, 29], and could thus participate to the control of dM HIV-1 infection. We have previously shown that decidual cell culture supernatants, which contain CCL3 and CCL4, partially inhibit the infection of decidual mononuclear cells by blocking the HIV-1 entry [4].

Our results show that dNK cells inhibit efficiently HIV-1 infection of dMs in vitro, and that this inhibition occurs before the complete establishment of infection. Contacts between dNK cells and dMs are important for an optimal control of HIV-1 infection, and IFN-γ is involved in the control of infection. Our study suggests that dNK cells could participate to the control of HIV-1 mother-to-child transmission in vivo. Studies are needed to determine if mucosal NK cells from the reproductive tract of non-pregnant women (FRT) are able to control HIV-1 sexual transmission. It will be also important to determine if an efficient NK cell response, such as the one in the decidua, could be induced in the FRT mucosae to prevent HIV-1 transmission. The study of the natural control of HIV-1 infection in the decidua give important information on the innate immune response to induce for the prevention of HIV-1 transmission.

## Conclusions

This study reveals that, besides their important role of builder in pregnancy, dNK cells can play a critical role in the control of HIV-1 infection at the materno-fetal interface and provides evidence that IFN-γ is involved. Our results highlight the role of innate immune determinants in the control of HIV-1 transmission.

## Methods

### Human decidual tissue collection and isolation of decidual mononuclear cells

Decidual tissues were obtained from healthy women undergoing voluntary termination of pregnancy during the first trimester (8–12 weeks of gestation) at Antoine Béclère Hospital (Clamart, France) or Pitié Salpêtrière Hospital (Paris, France). The tissues were minced and digested with 1 mg/mL collagenase IV (Sigma, St Quentin Fallavier, France) and 250U/mL recombinant DNase I (Roche, Meylan, France) for 45 min at 37 °C with agitation. The cell suspension was filtered successively through 100-, 70- and 40-µm pore-size sterile nylon cell strainers (BD Biosciences, Le pont de Claix, France). Mononuclear cells were isolated from the cell suspension by Ficoll gradient centrifugation using Lymphocyte Separation Medium (PAA, Les Mureaux, France). CD14^+^ cells (dMs) and CD56^+^ cells (dNK) were purified by positive selection with anti-CD14 and anti-CD56 magnetic beads (Miltenyi, Paris, France). In the decidual samples (n = 21), the mean of CD45^+^ cells represented 36 % from which 13 % were CD14^+^ and 62 % CD56^+^. After isolation, the mean of the number of cells obtained was 6.7 × 10^6^ for the CD14 + cells (out of 52 × 10^6^ total cell number) and 9.8 × 10^6^ for the CD56^+^ cells (out of 41 × 10^6^ total cell number). The purity of cells was checked by stainings and flow cytometry. dM and dNK cell purity was 93 % ± 3.8 % and 95 % ± 2 % respectively (mean + SEM) (Additional file 4: Fig S4). Contaminating cells were mainly CD45^neg^ cells in both dM and dNK cell samples. In order to verify that dNK cells (CD56^superbright^ CD9^+^) fraction did not contain any peripheral NK cells (CD56^dim/bright^ CD9^neg^), stainings were performed for each sample to analyse the expression of CD56 and CD9 by flow cytometry.

### HIV-1 isolate

Infections were performed with HIV-1_BaL_ (R5). HIV-1_BaL_ viral isolate was amplified for 11 days in PHA-stimulated PBMCs depleted of CD8^+^ cells, from three blood donors. The virus was concentrated by centrifugation on Vivaspin 100,000 kDa columns (Sartorius, Palaiseau, France) at 3000 rpm for 30 min, and was titrated on PBMCs.

### HIV-1 infection of purified dMs and coculture with dNK cells

dMs were incubated 1 h with HIV-1_BaL_ at an MOI of 10^−3^. dMs were then cultured alone at 10^6^ cells/mL or with dNK cells at 6 × 10^6^ cells/mL (ratio 1 dM:5 dNK). When dNK cells were added 3 or 16 h after infection, dNK cells were counted using trypan blue to exclude dead cells. During the 3 or 16 h after infection, dNK cells were stored at 4 °C and at 2 × 10^6^ cells/mL. In the double chamber coculture system, dMs were cultured in the top chamber and dNK cells in the bottom chamber, and dMs were cultured alone at 0.4 × 10^6^ cells/mL, or with dNK cells at 2x10^6^ cells/mL. Cells were cultured in HamF12 and DMEM glutamax (Gibco, Cergy Pontoise, France) supplemented with 15 % of fetal calf serum (PAA), penicillin (0.1 U/mL) and streptomycin (10^−8^ g/L). Human recombinant IL-15 (R&D systems, Lille, France) was added at 0.5 ng/mL in cocultures and in dM and dNK cell cultures. Viral production was measured every 3 or 4 days by the quantification of the HIV-1 p24 antigen (p24 Ag) in cell culture supernatants by ELISA (Zeptometrix, Franklin, Massachusetts). The percentage of inhibition was calculated as followed (Eq. (1)):

1 $$\frac{{[{\text{p}}24\,{\text{Ag}}] {\text{dM}} - [{\text{p}}24\,{\text{Ag}}] {\text{coculture}}}}{{[{\text{p}}24{\text{Ag}}]{\text{dM}}}} \times 100$$

The percentage of inhibition of the p24 Ag production has been calculated on day 20 post-infection as this time point is, for most samples, the peak of dM viral production. The calculation day has been chosen according to the p24 Ag production of dMs and not of cocultures, as for some samples no viral production was observed in cocultures.

### dM incubation with double chamber coculture supernatants

Uninfected double chamber coculture supernatants were harvested in the bottom chamber after 48 h of culture and cleared (5 min at 10,000 rpm). Supernatants were treated or not with an anti-IFN-γ antibody or an IgG isotype control (Becton–Dickinson, Le Pont de Claix, France) at 10μg/mL during one hour at room temperature (Fig. 5b). dMs from another sample infected with HIV-1_BaL_ at an MOI of 10^−3^ one hour before, were then incubated during 3 days with these supernatants. Viral production was measured every 3 or 4 days as previously described.

### Antibodies, reagents and flow cytometry

The purity of cells was determined by flow cytometry stainings with the following antibodies: CD45-Amcyan, CD14-Pacific Blue, CD3-PE TexasRed, CD4-PE Cy7, CD8-APC (Beckman coulter, Villepinte, France), CD56-A700 (Becton–Dickinson). The Fixable Viability Dye eFluor^®^ 780 (eBiosciences) was used to determine dNK cell viability. The following antibodies were used to analyse dNK cell phenotype: CD8-PB, CD85j-PE, NKp46-PE, NKp44-PE (Beckman Coulter), CD69-FITC, NKG2D-APC, NKp30-AF647 (Becton–Dickinson). The antibody CD119-PE (Becton–Dickinson) was used to analyse the expression of the IFN-γ receptor α-chain (CD119). Samples were analysed by flow cytometry with a LSRII 2-Blue 2-Violet 3-Red 5-Yelgr laser configuration (BD Biosciences) and FlowJo 9.1.3. software (Tristar).

### Cytokine quantification assay

Supernatants of infected dMs, infected and uninfected cocultures, infected and uninfected double chamber cocultures and dNK cells were harvested after 48 h of culture. Soluble factors were quantified by Luminex® assay (Cytokine human magnetic 25 plex panel, Invitrogen).

### Statistical analyses

Statistical analyses were performed using the GraphPad Prism software version 5.0f. The sign-rank test for paired data was used in all experiments except for the IFN-γ blocking experiment. To analyse the effect of the IFN-γ blocking in supernatants on dM viral production, a non-parametric permutation test for paired samples was used due to the limited sample size (4 donors).

### Ethics approval and consent to participate

All the women donors in this study provided their written informed consent. All experiments were performed in accordance with the approved guidelines and regulations. The Biomedical agency (No. PF508-013), “Assistance Publique des Hôpitaux de Paris” (No. VAL/2011/06-41/02) and the biomedical research committee of the Institut Pasteur, Paris, France (no. 2005.024) approved the study. The blood used for the viral isolate amplification on PBMCs was provided by “Établissement Français du Sang” (No. 12/EFS/134/No. HS2013-24916).

## Additional files

10.1186/s12977-016-0271-z Control of dM HIV-1 infection by dNK cells. dMs were infected with HIV-1_BaL_ at an MOI of 10-3. dNK cells were added or not to dMs before infection, 1, 3, 16 or 24 hours after infection, at a ratio 1 dM:5 dNK. Viral production was followed by the quantification of the p24 Ag in the supernatants. p24 Ag concentration in dM supernatants and in coculture supernatants is displayed over time for a representative donor.
10.1186/s12977-016-0271-z Role of NKG2D and CD85j in the control of HIV-1 infection. dMs were infected with HIV-1_BaL_ at an MOI of 10-3. dNK cells were added or not to dMs at a ratio 1 dM:5 dNK. dMs were treated with human fusion protein Fc-IgG or Fc-NKG2D or Fc-CD85j during 30 minutes after infection and before dNK cells were added. Fusion proteins were then added during the culture every 3 or 4 days. Viral production was followed by the quantification of the p24 Ag in the supernatants. The percentage of inhibition of the infection was calculated at day 19 post-infection. The medians of 4 samples are displayed. Each symbol represents one individual donor. Fusion protein Fc-IgG, Fc-NKG2D or Fc-CD85j (R&D systems) were used at 20µg/ml.
10.1186/s12977-016-0271-z CCL4 and CCL5 concentrations in the culture supernatants. CCL4 and CCL5 concentrations in 48h supernatants of infected dMs, infected double chamber cocultures, infected cocultures and dNK cells are depicted on the graph in pg/mL (9 donors, except for dNK cell supernatants, 6 donors). The medians are displayed. The sign-rank test for paired data was used. * p=0.031; ** p=0.004.
10.1186/s12977-016-0271-z Purity of dM and dNK cells. The purity of dNK cells (CD14- CD56+) and dMs (CD14+ CD56-) was checked by flow cytometry after cell isolation. A representative example is shown.
