# Supplementary figures and images for: NK cells control HIV‐1 infection of macrophages through soluble factors and cellular contacts in the human decidua
Source: Retrovirology. 2016 Jun 6;13:39. doi: 10.1186/s12977-016-0271-z (PMC4895978; doi:10.1186/s12977-016-0271-z)

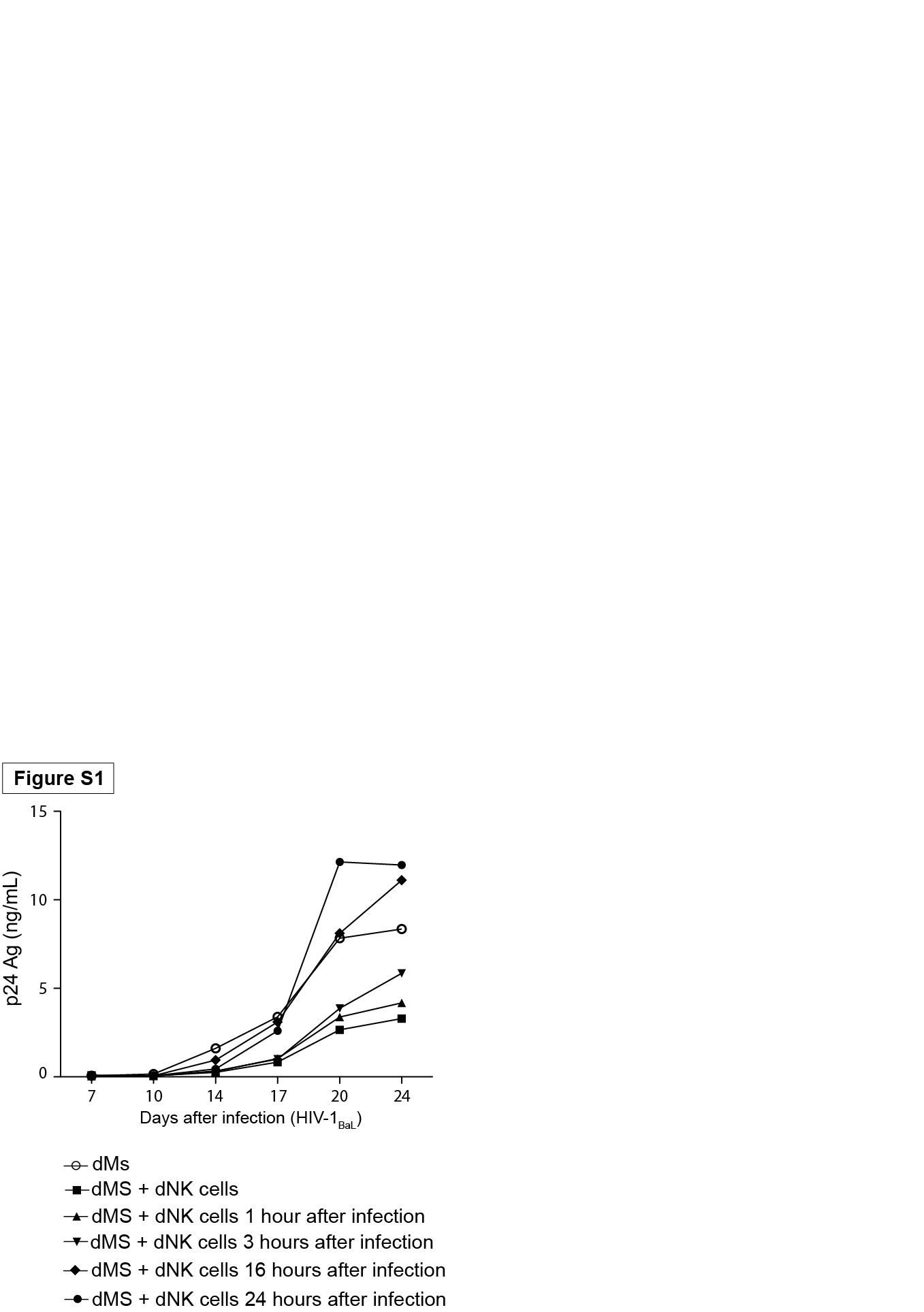

Supplement: Supplementary file 1 — 10.1186/s12977-016-0271-z Control of dM HIV-1 infection by dNK cells. dMs were infected with HIV-1BaL at an MOI of 10-3. dNK cells were added or not to dMs before infection, 1, 3, 16 or 24 hours after infection, at a ratio 1 dM:5 dNK. Viral production was followed by the quantification of the p24 Ag in the supernatants. p24 Ag concentration in dM supernatants and in coculture supernatants is displayed over time for a representative donor. [file 12977_2016_271_MOESM1_ESM.jpg]

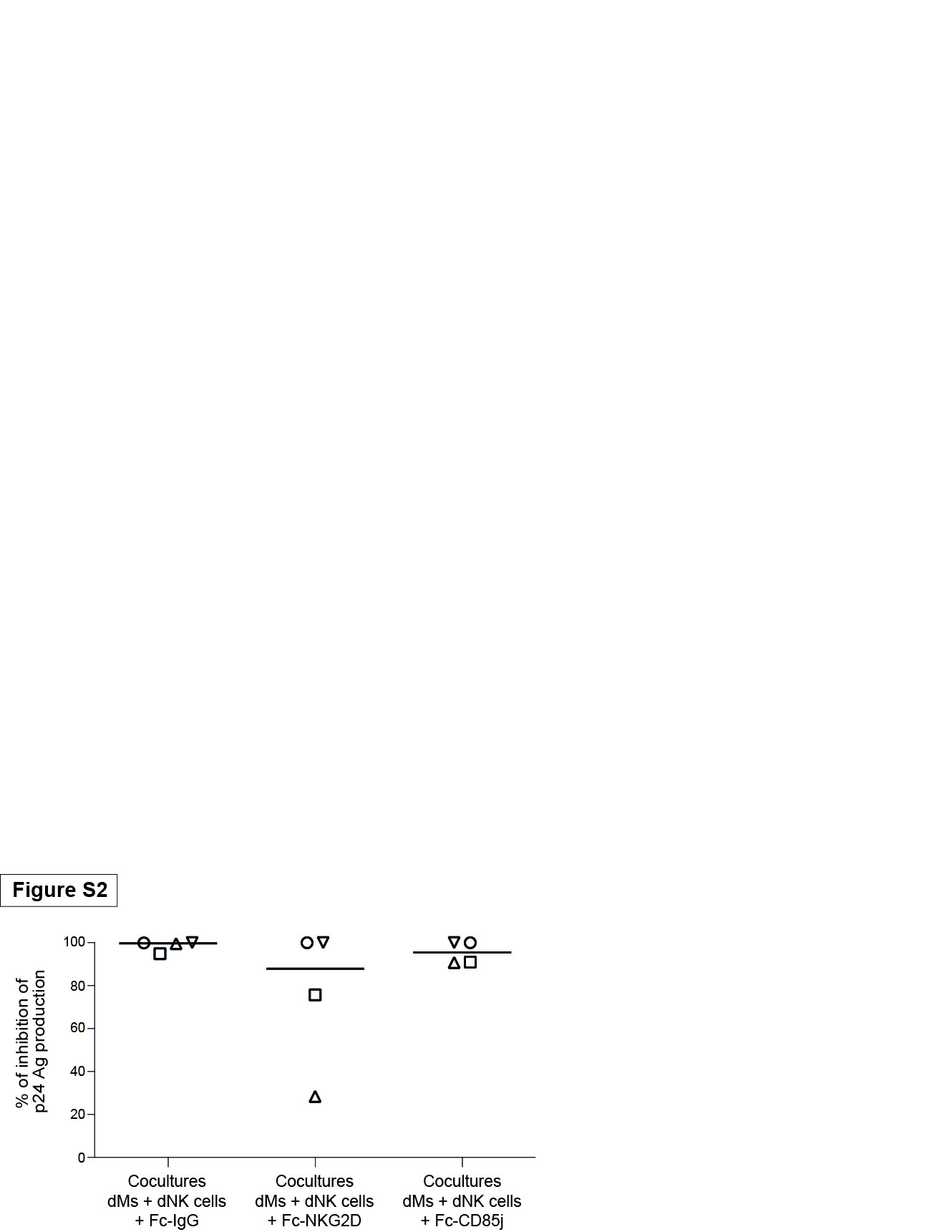

Supplement: Supplementary file 2 — 10.1186/s12977-016-0271-z Role of NKG2D and CD85j in the control of HIV-1 infection. dMs were infected with HIV-1BaL at an MOI of 10-3. dNK cells were added or not to dMs at a ratio 1 dM:5 dNK. dMs were treated with human fusion protein Fc-IgG or Fc-NKG2D or Fc-CD85j during 30 minutes after infection and before dNK cells were added. Fusion proteins were then added during the culture every 3 or 4 days. Viral production was followed by the quantification of the p24 Ag in the supernatants. The percentage of inhibition of the infection was calculated at day 19 post-infection. The medians of 4 samples are displayed. Each symbol represents one individual donor. Fusion protein Fc-IgG, Fc-NKG2D or Fc-CD85j (R&D systems) were used at 20µg/ml. [file 12977_2016_271_MOESM2_ESM.jpg]

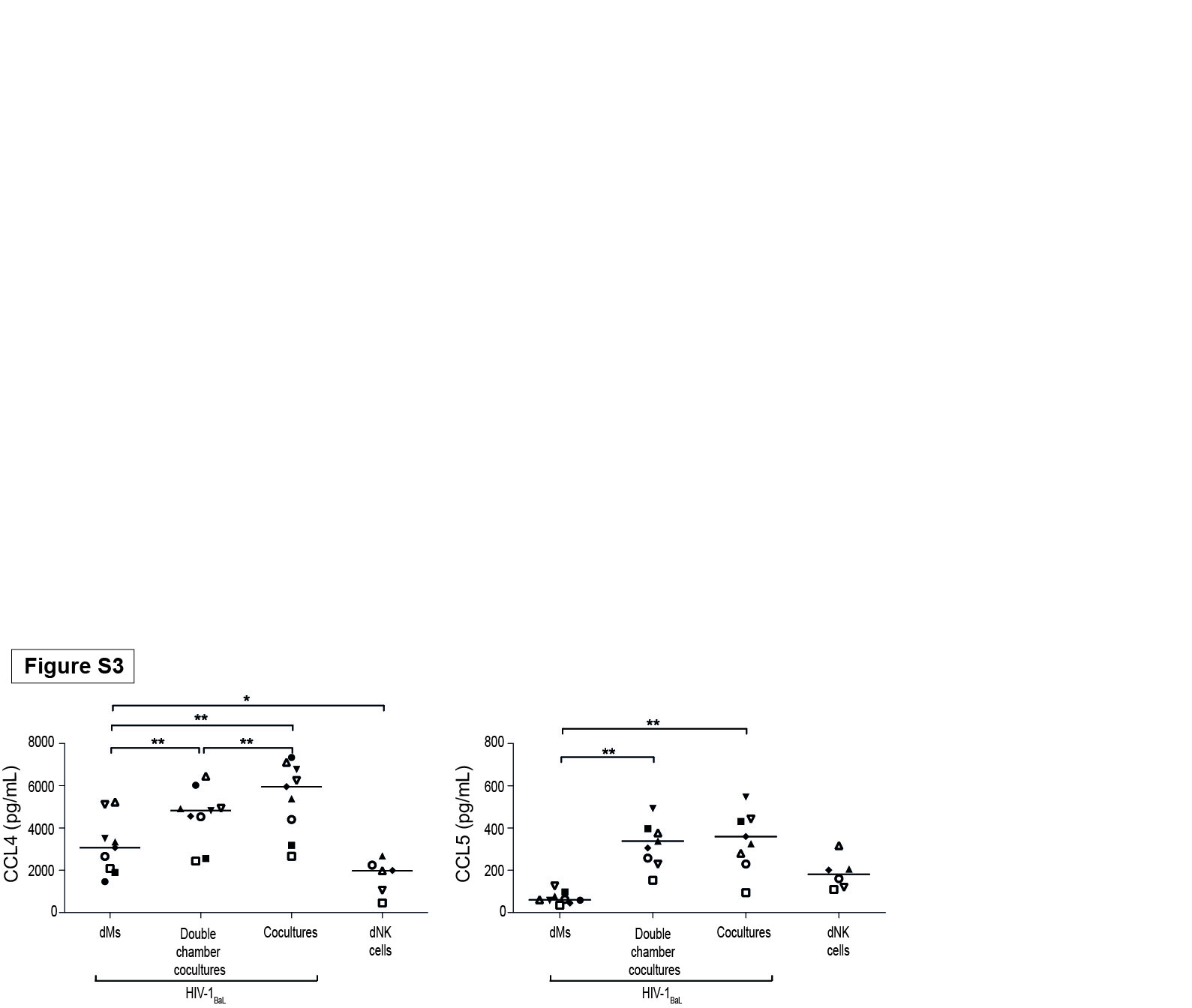

Supplement: Supplementary file 3 — 10.1186/s12977-016-0271-z CCL4 and CCL5 concentrations in the culture supernatants. CCL4 and CCL5 concentrations in 48h supernatants of infected dMs, infected double chamber cocultures, infected cocultures and dNK cells are depicted on the graph in pg/mL (9 donors, except for dNK cell supernatants, 6 donors). The medians are displayed. The sign-rank test for paired data was used. * p=0.031; ** p=0.004. [file 12977_2016_271_MOESM3_ESM.jpg]

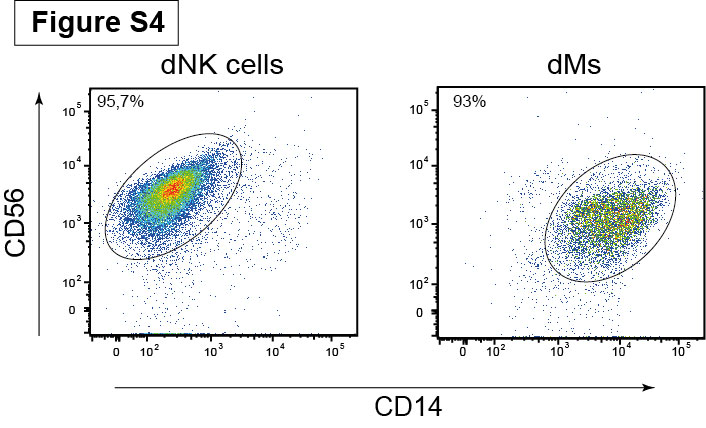

Supplement: Supplementary file 4 — 10.1186/s12977-016-0271-z Purity of dM and dNK cells. The purity of dNK cells (CD14- CD56+) and dMs (CD14+ CD56-) was checked by flow cytometry after cell isolation. A representative example is shown. [file 12977_2016_271_MOESM4_ESM.jpg]
